# Endoscopic main duct stenting in refractory postoperative pancreatic fistula after distal pancreatectomy – a friend or a foe?

**DOI:** 10.1186/s12893-023-02233-x

**Published:** 2024-01-24

**Authors:** Stefan Linder, Marcus Holmberg, Louiza Agopian-Dahlenmark, Helena Zhao, Johan Hardvik Åkerström, Ernesto Sparrelid, Poya Ghorbani

**Affiliations:** 1 Division of Surgery, Department of Clinical Science, Intervention and Technology, Karolinska Institutet, Stockholm, Sweden; 2 Karolinska University Hospital, Stockholm, Sweden; 3 Department of Surgery, Capio St Görans Hospital, Stockholm, Sweden; 4 Division of Surgery and urology, Mälarsjukhuset, Eskilstuna, Sweden; 5 Upper Gastrointestinal Surgery, Department of Molecular medicine and Surgery, Karolinska Institutet, Stockholm, Sweden

**Keywords:** Distal pancreatectomy, Postoperative pancreatic fistula, Risk factor, Endoscopic therapy, Complications

## Abstract

**Background:**

Clinically relevant (CR) postoperative pancreatic fistula (POPF) after distal pancreatectomy (DP) are common. Endoscopic treatment (ET) has only scarcely been explored. The aim of this study was to evaluate risk factors for CR POPF after DP and the efficacy of ET in adjunct to standard therapy.

**Methods:**

Consecutive patients without previous pancreatic surgery who underwent DP between 2011 and 2020 were evaluated, analyzing risk factors for CR POPF. The choice and performance of ET, main pancreatic duct (MPD) stenting, was not standardized. Healing time and complications after ET were registered.

**Results:**

406 patients underwent DP, CR POPF occurred in 29.6%. ET was performed in 17 patients 27 days (median) after index surgery. Risk for CR POPF was increased in ASA-PS 1–2 patients, MPD ≤ 3 mm, procedure time ≥ 3 h, and CRP ≥ 180 on postoperative day 3. POPF resolved with standard treatment after 32 days and 59 days in the ET group (p < 0.001). There was one mortality in the ET-group (not procedure related). Mild post-ERCP pancreatitis occurred in three patients.

**Conclusions:**

CR POPF is common after DP. Long operating time, a narrow MPD, low ASA score, and high postoperative CRP were risk factors for CR POPF. ET was not beneficial but proper evaluation was not possible due to few patients and non-standardized treatment. Complications after ET appeared mild.

## Background

Distal pancreatectomy (DP) with or without splenectomy is the standard operative procedure for malignant or benign diseases in the body and the tail of the pancreas [1, 2]. Clinically relevant (CR) postoperative pancreatic fistula (POPF) after DP is a fairly common complication reported in 12–30% of patients undergoing DP [3–7].

Morbidity after DP remains high (40-60%), however not reflected in mortality rates which are considered to be low (0–3%) [5, 6, 8–10]. POPF is the main contributor to a negative outcome after DP, including fluid collections, abscess formation, sepsis, delayed gastric emptying (DGE), formation of pseudoaneurysm and post-pancreatectomy hemorrhage (PPH) [11, 12].

Several mitigation strategies for POPF after DP have been described and more are under evaluation. However, the results are ambiguous without any consistent advantage leading to a universal change in standard of care [3, 11, 13–17]. Endoscopic stenting of the main pancreatic duct (MPD) preoperatively or intraoperatively has not convincingly reduced the rate of POPF after DP or enucleation techniques [18, 19]. Currently, the role of preoperative endoscopic injection of botulinum toxin into the sphincter of Oddi to prevent POPF is under investigation [20].

POPF usually resolves with standard treatment, i.e., prolonged and repeated drainage, and antibiotics [9, 21]. Analogous to endoscopic treatment (ET) of biliary leakage with sphincterotomy and stents [22], endoscopic pancreatic sphincterotomy (EPS) alone or together with stenting of the MPD has been used in the treatment of pancreatic injuries, acute pancreatitis (disconnected pancreatic duct syndrome), and chronic pancreatitis with fistula [23, 24]. It has been suggested that high pressure in the MPD may promote stump leakage [8]. Thus, the placement of a stent in the MPD could facilitate fistula resolving. However, this treatment adjunct has only been scarcely evaluated, and so far, without any promising results [25–28].

The aim of the present study was to further evaluate EPS and endoscopic stenting of the MPD in adjunct to conservative treatment in CR POPF after DP with regards to time to clinical healing. Furthermore, risk factors for CR POPF and possible differences between patients with CR POPF receiving endoscopic therapy or not were evaluated as well as complications after these interventions.

## Methods

This retrospective single center study was approved by the Ethical Committee Stockholm (registration number: DNr 2020/05238) and performed following the Strengthening the Reporting of Observational Studies in Epidemiology guidelines [29].

### Study population and design

All consecutive adult patients (age ≥ 18 years) who underwent an elective DP with or without splenectomy for any indication at Karolinska University Hospital (January 1, 2011 – December 31, 2020) were included. Patients who previously had undergone any type of pancreatic surgery, pre- or intra-operative ET or had missing laboratory data which impaired the assessment of POPF were excluded. The subgroups of patients without CR POPF (no CR POPF group) and those with (CR POPF group) were analyzed. The latter group was divided into those who underwent standard treatment (standard/no ET group) or ET (ET group).

### Baseline characteristics - patient factors

Registration Included sex, age, body mass index (BMI), indication for surgery (histological diagnosis, not malignant or malignant), treatment with neoadjuvant chemotherapy, smoking status (never, previous, current) and American Society of Anesthesiologists – Physical Status (ASA-PS) classification [30]. Comorbidity was defined according to the adaptation of Charlson comorbidity index for register-based studies [31].

### Baseline characteristics - perioperative variables

Data was collected regarding date of surgery, surgical technique (open or minimal invasive), and preservation of the spleen. Duration of surgery (< 3 h, 3-4 h, or > 4 h) and blood loss < 300 ml, 300 − 100 ml, or > 1000 ml) and the pancreatic remnant closure technique (stapling, hand sewn technique, or a combination of these methods) were also registered. As a rule, no mitigation strategy was used to reduce POPF rate. Extended resection was defined according to the International Study Group for Pancreatic Surgery (ISGPS) (extra-pancreatic organ resection or vascular resection) [32]. The level of resection line in relation to the portal vein/superior mesenteric vein (PV/SMV) was registered, as “pancreatic right of SMV” if the transection line was to the right of the SMV/PV. The size (diameter) of MPD (≤ 3 mm or > 3 mm) was estimated on preoperative computerized tomography (CT) or measured on the operative specimen. The pancreatic texture was based on intraoperative assessment and graded as soft, intermediate or hard [33].

### Variables related to the treatment of POPF

Standard treatment in the CR POPF group comprised of prolonged duration of routinely placed intraabdominal drains. When required, additional drains were inserted guided by ultrasonography or CT. Antibiotics were solely administered on suspicion of infection and the duration of therapy was recorded. Somatostatin analogues were not part of the routine treatment and used on the discretion of the responsible surgeon. Patient selection for ET in the CR POPF group was not standardized. ET consisted of ERCP with or without EPS, but always stent placement in the MPD, sometimes also passing the transection surface of the pancreas. In selected patients, endoscopic ultrasound guided drainage by double pigtail stents or lumen apposing metal stents were used (between stomach and fluid collections). When required, biliary endoscopic sphincterotomy was performed, sometimes also inserting a biliary stent. It was noted on which postoperative day (POD) ET was preformed, complications related to ET, days until the POPF resolved after ET and the resection. POPF was defined as resolved when one of the following criteria was met: the last drain was removed without signs of recurrence, no contrast leakage during ERCP or when the radiologist determined no leak on CT [21]. The MPD stent was removed within two months after closure of the POPF.

### Postoperative outcomes

POPF was graded according to the ISGPF definition as biochemical leakage, grade B or grade C (CR POPF) [7]. Patients with biochemical leakage were registered as no POPF. The ISPGS definitions of DGE and PPH were used [34, 35]. Post-pancreatectomy acute pancreatitis (PPAP) was also defined according to ISGPS [36]. The definition of ERCP-related complications followed the guidelines by the European Society of Gastrointestinal Endoscopy, post-ERCP pancreatitis, cholangitis, bleeding, and perforation [37]. Complications were graded according to the Clavien-Dindo classification of the surgical complications [38]. Specifically, deep infection, wound dehiscence, and re-laparotomy were recorded. Additional drainage was sometimes used in patients without POPF. Angiographic intervention was the primary tool in patients with PPH. The frequency of CT use within the first postoperative week was noted. C-reactive protein (CRP) (mg/L), amylase in serum (µkat/L) and drains (µkat/L) were analyzed POD 1, POD 2, and POD 3 (often daily during the hospitalization). The institutional upper limit for normal serum amylase activity was 1.15 µ-kat/L (equivalent to 69 IU/L). In the ET group these values were also registered as maximum values during the first postoperative week, pre-ET, and post-ET.

Total length of hospital stay, stay in high dependency unit and frequency of stay in intensive care unit were recorded. Length of hospital stay was calculated as total inpatient days from index surgery to the time of discharge from hospital including rehospitalization for related symptoms or adverse events.

### Statistical analyses

All statistical analyses were performed in R version 4.0.2 (Vienna, Austria. 2020). Covariates for various groups were compared using Wilcoxon rank sum test or Kruskal-Wallis rank sum test for continuous covariates and Chi-square test (or Fisher’s exact test when appropriate) for categorical variables, and presented either as medians and interquartile ranges (IQR) or percentages and frequencies, respectively.

Risk factors for POPF were analyzed by binary logistic regression analysis. Covariates with a significance level < 20% (p < 0.2) in univariable analysis were considered relevant and further explored in a multivariable model using backward stepwise selection. We accepted a saturated initial model. Odds ratios (ORs) for CR POPF with associated 95% confidence intervals (CIs) were calculated. In all analyses the critical level of significance was set to 5% (p < 0.05).

## Results

### Demographic data, surgical techniques, postoperative treatment and clinicopathological variables

**Fig. 1:**
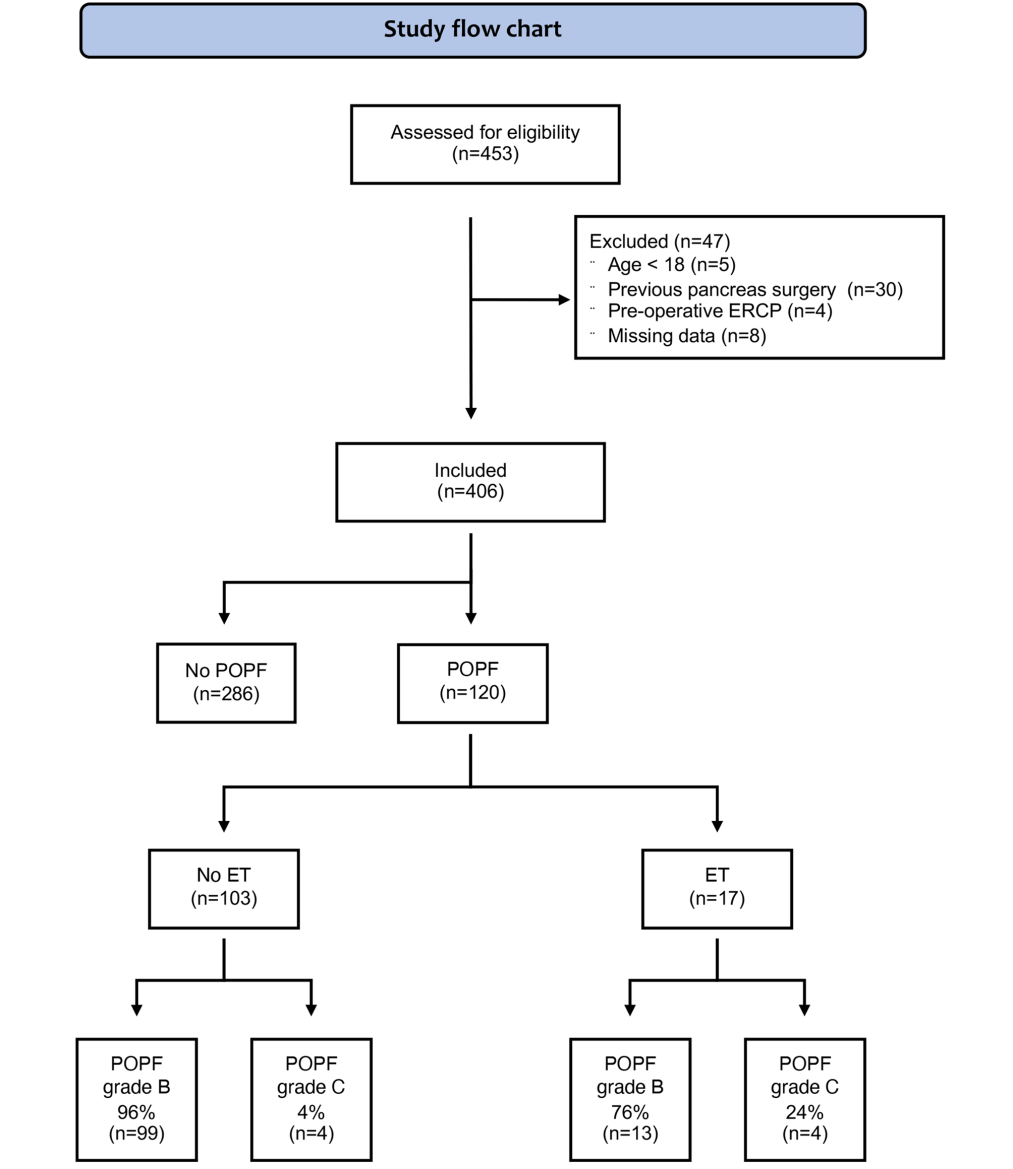
Flowchart for the study. Abbreviations: POPF – post operative pancreatic fistula; ET – endoscopic treatment; grade B or C – POPF grade B or C according to ISGPS.

A total of 453 patients underwent DP, of these 47 patients were excluded. Thus, the final cohort consisted of 406 patients (Fig. 1). The rate of CR POPF was 29.6% (120/406). ET was performed in 17 patients (14.2%). Baseline characteristics for patients with no CR POPF and with CR POPF receiving standard treatment or ET are demonstrated in Table 1. In the CR POPF group ASA-PS was lower, spleen preservation more common, procedure time longer, and MPD diameter smaller.

**Table 1:** Baseline characteristics

Variable	Overall N = 406^*1*^	No POPF n = 286^*1*^	POPF n = 120^*1*^	p-value^*2*^		POPF		p-value^*2*^
						No ET n = 103^*1*^	ET n = 17^*1*^	
**Sex**				0.370				0.573
Female	227 (56)	164 (57)	63 (52)			53 (51)	10 (59)	
Male	179 (44)	122 (43)	57 (48)			50 (49)	7 (41)	
**Age**	67 (56–73)	68 (58–74)	64 (52–71)	**0.015**		64 (52–72)	64 (55–71)	0.922
**ASA**				**0.042**				0.258
1 to 2	251 (63)	166 (60)	85 (71)			75 (73)	10 (59)	
3 to 4	145 (37)	110 (40)	35 (29)			28 (27)	7 (41)	
**CCI**				0.909				0.304
1 to 2	343 (84)	242 (85)	101 (84)			85 (83)	16 (94)	
3 to 5	63 (16)	44 (15)	19 (16)			18 (17)	1 (5.9)	
**BMI**	26 (23–29)	25 (23–29)	26 (24–30)	0.104		26 (24–29)	28 (25–32)	0.154
**Smoking**				0.368				0.745
Never	236 (59)	165 (59)	71 (59)			61 (59)	10 (59)	
Previous	105 (26)	70 (25)	35 (29)			29 (28)	6 (35)	
Current	61 (15)	47 (17)	14 (12)			13 (13)	1 (5.9)	
**Indication**				0.093				0.105
Not malignant	167 (41)	125 (44)	42 (35)			39 (38)	3 (18)	
Malignant	237 (59)	159 (56)	78 (65)			64 (62)	14 (82)	
**Neo-adjuvant therapy**	13 (3.2)	9 (3.1)	4 (3.3)	> 0.999		99 (96)	17 (100)	1.000
**Procedure**				**0.034**				0.346
Open	345 (85)	250 (87)	95 (79)			83 (81)	12 (71)	
Minimally invasive	61 (15)	36 (13)	25 (21)			20 (19)	5 (29)	
**Spleen preserving**	27 (6.7)	17 (5.9)	10 (8.3)	0.378		6 (5.8)	4 (24)	0.034
**Procedure time**				**0.004**				0.188
< 3 h	152 (38)	121 (43)	31 (26)			29 (28)	2 (12)	
3-4 h	112 (28)	70 (25)	42 (35)			33 (32)	9 (53)	
> 4 h	135 (34)	88 (32)	47 (39)			41 (40)	6 (35)	
**Intra-op blood loss**				0.163				**0.007**
< 300ml	224 (56)	158 (56)	66 (56)			61 (61)	5 (29)	
300-1000ml	141 (35)	105 (37)	36 (31)			30 (30)	6 (35)	
> 1000ml	36 (9.0)	21 (7.4)	15 (13)			9 (9.0)	6 (35)	
**Stump closure**				0.893				0.697
Stapler	198 (49)	139 (49)	59 (49)			52 (50)	7 (41)	
Suture	69 (17)	47 (17)	22 (18)			19 (18)	3 (18)	
Stapler and Suture	136 (34)	97 (34)	39 (32)			32 (31)	7 (41)	
**Gland texture**				0.365				0.624
Soft	135 (33)	91 (32)	44 (37)			38 (37)	6 (35)	
Intermediate	198 (49)	146 (51)	52 (43)			43 (42)	9 (53)	
Hard	73 (18)	49 (17)	24 (20)			22 (21)	2 (12)	
**Duct dimension**				**0.018**				1.000
≤ 3	250 (87)	164 (83)	86 (93)			75 (93)	11 (100)	
> 3	39 (13)	33 (17)	6 (6.5)			6 (7.4)	0 (0.0)	
**Extended resection**								
Pancreatic (right of SMV)	39 (9.6)	28 (9.8)	11 (9.2)	0.846		7 (6.8)	4 (24)	**0.049**
Extra-pancreatic (organ)	98 (24)	66 (23)	32 (27)	0.441		27 (26)	5 (29)	0.773
Vascular^3^	16 (3.9)	8 (2.8)	8 (6.7)	0.091		5 (4.9)	3 (18)	0.085

^1^ n (%); Median (25-75%).

^2^ Pearson’s c2 test; Wilcoxon rank sum test; Fisher’s exact test.

^3^ Arterial resection in total n = 3.

Extended resection according to ISGPS = extra-pancreatic (organ) + vascular.

POPF, postoperative pancreatic fistula; ET, endoscopic treatment; BMI, body mass index; SMV, superior mesenteric vein.

### Postoperative Complications and healing time

**Fig. 2:**
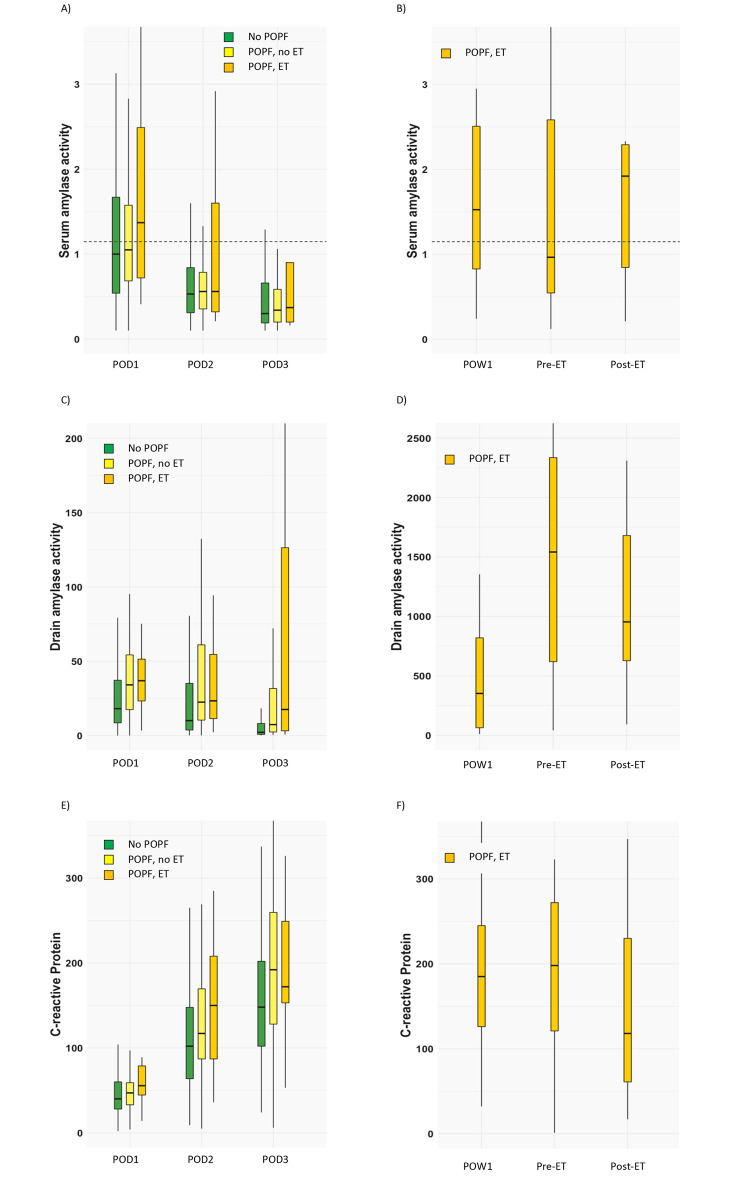
Laboratory dynamics for patients not developing post operative pancreatic fistula (POPF) and for patients with POPF managed with endoscopic treatment (ET) or not. The dotted line in **A** and **B** represents the institutional upper normal limit. The dynamics comprise post-operative day (POD) 1–3 for figures **A**, **C** and **E**, as well as maximum values first post-operative week (POW1), pre- and post-ET for figures **B**, **D** and **F**. **(A)** Serum amylase activity POD1-3 **(B)** Max values for serum amylase activity POW1, pre-ET and post-ET. **(C)** Drain amylase activity POD1-3 **(D)** Max values for drain amylase activity POW1, pre-ET and post-ET. Note the change in the y-axis. **(E)** C-reactive Protein POD1-3 **(F)** Max values for C-reactive Protein POW1, pre-ET and post-ET.

Drain amylase was higher in CR POPF patients POD 1–3 than among those without (Fig. 2) (Table 2). The rate of postoperative complications is described in Table 3. CR POPF increased the complication rates, length of hospital stay, stay in intensive care unit, and number of medical/interventional procedures. PPH was more common in CR POPF patients (p = 0.002). CT within the first week was performed in 16% of patients with CR POPF as compared to 4.3% among those without (p < 0.001). Additional drains were inserted in 52% of patients with CR POPF while only 5.3% of patients without CR POPF required drainage (p < 0.001).

**Table 2:** Laboratory characteristics

Variable	Overall N = 406^1^	No POPF n = 286^1^	POPF n = 120^1^	p-value^2^	POPF		p-value^2^
					No ET n = 103^1^	ET n = 17^1^	
**Serum amylase**							
POD1	1.0 (0.6–1.6)	1.0 (0.6–1.7)	1.1 (0.7–1.6)	0.372	1.0 (0.7–1.6)	1.4 (0.7–2.5)	0.244
POD2	0.5 (0.3–0.8)	0.5 (0.3–0.8)	0.6 (0.3–0.8)	0.182	0.6 (0.3–0.8)	0.6 (0.3–1.6)	0.513
POD3	0.3 (0.2–0.6)	0.3 (0.2–0.5)	0.3 (0.2–0.6)	0.086	0.3 (0.2–0.5)	0.4 (0.2–0.7)	0.540
Elevated either POD1-3	181 (47)	124 (46)	57 (49)	0.681	48 (48)	9 (53)	0.706
Elevated sustained > 48hr	50 (19)	32 (18)	18 (22)	0.395	12 (12)	6 (35)	0.073
**Drain amylase**							
POD1	22 (10–43)	18 (8.5–38)	34 (18–54)	**< 0.001**	34 (17–54)	37 (23–51)	0.703
POD2	14 (5.0–40)	10 (3.7–35)	23 (10–61)	**< 0.001**	23 (10–61)	23 (11–55)	0.807
POD3	2.9 (1.1–13)	2.1 (0.8–8.1)	8.5 (2.4–38)	**< 0.001**	7.4 (2.4–32)	18 (3.2–126)	0.167
**C-reactive Protein**							
POD1	43 (29–60)	40 (28–60)	48 (34–61)	0.061	47 (33–59)	56 (48–79)	0.109
POD2	108 (68–160)	102 (64–147)	120 (87–177)	**0.002**	117 (86–169)	150 (117–208)	0.246
POD3	160 (105–220)	148 (102–202)	192 (137–259)	**< 0.001**	192 (124–256)	172 (153–249)	0.955

^1^Median (25-75%); n (%).

^2^Pearson’s Chi-squared test; Wilcoxon rank sum test; Fisher’s exact test.

POPF, postoperative pancreatic fistula; ET, endoscopic treatment; POD postoperative day.

**Table 3:** Complications, management, and outcomes

Variable	Overall N = 406^1^	No POPF n = 286^1^	POPF n = 120^1^	p-value^2^	POPF		p-value^2^
					No ET n = 103^1^	ET n = 17^1^	
**Hospital stay (days)**	9 (7–14)	9 (7–12)	12 (8–18)	**< 0.001**	11 (8–15)	24 (15–51)	**< 0.001**
**Re-admission (%)**	94 (23)	35 (12)	59 (50)	**< 0.001**	47 (46)	12 (75)	**0.031**
**Intermediary stay (%)**	334 (82)	237 (83)	97 (81)	0.574	81 (79)	16 (94)	0.189
**Intermediary days**	2 (2–4)	2 (2–4)	2 (2–5)	0.717	2 (1–3)	5 (2–7)	**0.004**
**ICU-stay 24 hr (%)**	19 (4.7)	8 (2.8)	11 (9.2)	**0.006**	7 (6.8)	4 (24)	**0.049**
**Drain duration (days)**	8 (6–20)	7 (5–9)	32 (20–50)	**< 0.001**	30 (20–48)	41 (23–91)	0.147
**Additional drain (%)**	78 (19)	15 (5.3)	63 (52)	**< 0.001**	51 (50)	12 (71)	0.107
**Antibiotics**	238 (59)	125 (44)	113 (94)	**< 0.001**	96 (93)	17 (100)	0.591
**Antibiotics (days)**	13 (8–23)	10 (6–14)	21 (14–34)	**< 0.001**	20 (13–32)	30 (18–66)	**0.017**
**Deep infection (%)**	101 (25)	27 (9.5)	74 (62)	**< 0.001**	58 (56)	16 (94)	**0.003**
**Re-laparotomy (%)**	25 (6.2)	9 (3.2)	16 (13)	**< 0.001**	13 (13)	3 (18)	0.699
**Wound dehiscence (%)**	5 (1.2)	4 (1.4)	1 (0.9)	1.000	4 (3.9)	1 (5.9)	0.541
**Angio-intervention (%)**	6 (1.5)	1 (0.4)	5 (4.2)	**0.010**	1 (1.0)	0 (0)	1.000
**CT within first week (%)**	29 (7.8)	11 (4.3)	18 (16)	**< 0.001**	14 (15)	4 (24)	0.469
Pancreatitis (%)	2 (6.9)	1 (9.1)	1 (5.6)	1.000	0 (0)	1 (25)	0.222
**PPAP (%)**				1.000			1.000
No	330 (100)	228 (100)	102 (100)		89 (100)	13 (100)	
Yes	1 (0.3)	1 (0.4)	0 (0)		0 (0)	0 (0)	
**POPF (%)**				**< 0.001**			**0.014**
No or A	285 (70)	285 (100)	0 (0)		0 (0)	0 (0)	
B	112 (28)	0 (0)	112 (93)		99 (96)	13 (76)	
C	8 (2.0)	0 (0)	8 (6.7)		4 (3.9)	4 (24)	
**PPH (%)**				**0.002**			**0.005**
No or A	381 (94)	274 (96)	107 (89)		95 (92)	12 (71)	
B	17 (4.2)	10 (3.5)	7 (5.8)		6 (5.8)	1 (5.9)	
C	7 (1.7)	1 (0.4)	6 (5.0)		2 (1.9)	4 (24)	
**DGE (%)**				0.828			**0.019**
No or A	397 (98)	280 (98)	117 (98)		102 (99)	15 (88)	
B	4 (1.0)	3 (1.1)	1 (0.8)		1 (1.0)	0 (0)	
C	4 (1.0)	2 (0.7)	2 (1.7)		0 (0)	2 (12)	
**Clavien-Dindo grade**				**< 0.001**			**< 0.001**
0–2	303 (75)	247 (87)	56 (47)		56 (54)	0 (0)	
3	83 (20)	30 (11)	53 (44)		40 (39)	13 (76)	
4	16 (4.0)	7 (2.5)	9 (7.5)		6 (5.8)	3 (18)	
5	3 (0.7)	1 (0.4)	2 (1.7)		1 (1.0)	1 (5.9)	

^1^Median (25-75%); n (%).

^2^Wilcoxon rank sum test; Pearson’s Chi-squared test; Fisher’s exact test.

POPF, postoperative pancreatic fistula; ET, endoscopic treatment; ICU, intensive care unit; CT, computerized tomography; PPAP, post-pancreatectomy acute pancreatitis; PPH, post-pancreatectomy hemorrhage; DGE, delayed gastric emptying.

The median time for POPF to resolve after resection with standard treatment was 32 days (IQR 21–49) and in the ET group closure occurred after 59 days (IQR 42–156) (p < 0.001). Once ET was performed the median time for POPF to resolve after stenting was 34 days (IQR 15–116).

### Risk factors for CR POPF

The risk for CR POPF was decreased in ASA-PS 3–4 patients (OR 0.44, 95% CI 0.24–0.77, p = 0.005), increased in MPD ≤ 3 mm (OR 3.08, 95% CI 1.25–8.78, p = 0.022), procedure time ≥ 3 h (OR 2.15, 95% CI 1.24–3.80, p = 0.008), and CRP ≥ 180 on POD3 (OR 2,26, 95% CI 1.28–4.03, p = 0.005).

### ET-specific findings

The median time from index surgery to first ET was 27 days (IQR 15–37). Baseline characteristics and complications are shown in Tables 1 and 3. In the ET group intraoperative blood loss was larger and transection right of the SMV was more common as compared to the standard group. PPH was more frequent in the ET group (p = 0.005) as were many other variables related to postoperative complications. Higher drain amylase levels were encountered in the ET group during the first post-operative week, prior to ERCP, and lower levels were seen after the procedure (Fig. 2) (Table 2).

**Fig. 3:**
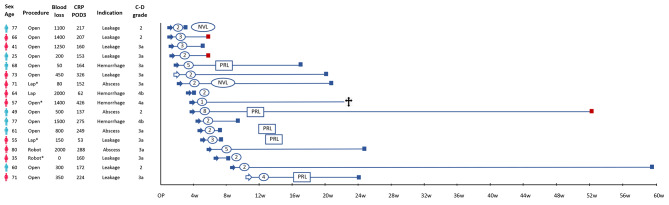
Presentation of and timeline for patients managed with endoscopic treatment (ET). Blue figure indicates male, red female. Procedure marked with * indicates spleen preserving procedure; Blue arrow indicates time point for first ET, filled arrow indicates ET with endoscopic pancreatic sphincterotomy (EPS) and stent, hollow arrow indicates ET by stent without EPS; Square indicates when POPF was considered healed, blue color without complication, red with complication; Black cross indicates fatal outcome; Encircled number indicates numbers of ET´s (including stent removal). Abbreviations: POPF – post operative pancreatic fistula; CRP – C-reactive protein; POD – post operative day; C-D grade – complication grade according to Clavien-Dindo pre-ET; NVL – non-visualized leakage; PRL – stent passed resection line

The timeline for patients managed with ET including detailed information regarding the procedures is presented in Fig. 3. Four patients underwent dilation of the MPD in addition to stenting. Endoscopic ultrasound-based drainage was used in two cases, one by double pigtail and in one a lumen apposing metal stent (HotAxios) was introduced. Biliary endoscopic sphincterotomy was performed in nine patients, four also received biliary stent. Additional percutaneous drainage was used in 12 patients (70.6%).

Mild post-ERCP pancreatitis after ET occurred in three patients, there were no cases of cholangitis, perforation, or hemorrhage. There was one mortality which was not related to the ET.

## Discussion

The present study including 120 patients with CR POPF indicates that ET (performed in 14%), complementary to standard treatment does not seem to be superior to standard treatment alone, healing time (59 days vs. 32 days) and hospital stay (24 vs. 11 days). ET seems safe but after ET the time to POPF resolution was long (34 days). Operating time ≥ 3 h, MPD diameter ≤ 3 mm, ASA 1–2, and a CRP ≥ 180 on POD 3 were independent risk factors for CR POPF.

Watanabe et al. [25] demonstrated a similar hospital stay with or without ET (40 days) and the mean healing time after ET was 46 days in 11 patients. Reddymasu et al. [28] reported healing in eight patients 44–379 days after ET. Goasguen et al. [26] evaluated ten patients (including two enucleations) who underwent ET, experiencing a POPF resolution after 1–12 days. Similarly, Grobmyer et al. [27] analyzed eight patients treated with ET and a healing was achieved after 32–84 days. Apparently, there is often a long healing time of POPF after ET with a wide variation in duration. The reason for this is not clear from current data based on small retrospective studies. In contrast to the treatment of biliary leakage, a possible downstream control with a reduction of MPD pressure after ET does not seem to be the obvious mechanism solving the problem [8, 22].

In the present study the selection to perform ET (14%) and time to intervention (8–79 days) was not standardized. In previous studies the frequency of ET in studied cohorts was 29–62% or not reported, the time duration from DP to performing ET has also varied (12–120 days) [25–28]. Thus, all studies suffer from a selection bias and a lack of uniform management. As in the present series, patients with a more severe condition may probably have been offered ET more frequently. In our study the rate of CT within the first postoperative week was 24% in the ET group corresponding with the need for CT in the presence of suspected complications as proposed in a recent randomized controlled trial [39].

Like other studies investigating the role of ET there are methodological variations regarding numbers of stents, dimensions, length of stents, and indications for repeating ET [25–28]. As in the present study, ET has sometimes not included EPS [26] and in one study stents were always inserted without EPS [28]. Moreover, as reported by others ET may also aim at draining collections, thus passing the resection line (5/17 in the present series) [25]. In the present series ET also included endoscopic ultrasound and drainage by double pigtail or lumen apposing metal stents in two patients, not obviously contributing to POPF resolution. A high rate of technical and clinical success has been reported using drainage by endoscopic ultrasound alone but similar to ERCP-based ET, the time until resolution is long [40].

Mild post-ERCP pancreatitis was the only type of complication after ET in the present series and occurred in 17.6% of the patients subjected to this procedure. Our study is small and comparison with the literature is difficult, systematic reviews have reported a rate of post-ERCP pancreatitis in unselected patients between 3.5% and 9.7% [41]. In comparison, in our cohort the aim was a pancreatic intervention in an already vulnerable situation. It may also be difficult to distinguish between what was a true complication of the ET and the development of the primary event. No complications or no “serious complications” after ET were reported by others [25–28]. Thus, it may appear that the complication rate after ET seem low and mild in character not adding an obvious burden to the already serious condition. Multiple procedures were often needed, in our series 53% underwent repeated ET. The rate of repeated ET has ranged from 10 to 25% in other series [25–28].

The rate of CR POPF after DP in our study was 29.6% which is in the higher range of previous reports [1, 3–5, 8, 11, 42]. Most resections in the present series were performed by open surgery and in accordance with the ISGPS guidelines, and as demonstrated in a recent meta-analysis, the surgical approach did not affect the frequency of CR POPF in our study [14, 43]. In conformity with others, we found no influence on the rate of CR POPF by the choice of closing method [3, 10]. In line with other studies, extended resection was not a risk factor for CR POPF in the present series [3, 4, 10, 21, 44]. There are conflicting results regarding the level of transection line and incidence of POPF; some reports indicate an increased risk dividing on either side of the pancreatic neck [4, 45, 46]. As reported by others, in the present study a transection right of the pancreatic neck was not related with CR POPF [3, 6, 47], but was indeed associated with ET.

Our study identified ASA-PS 1–2 status, MPD ≤ 3 mm, procedure time ≥ 3 h, and CRP ≥ 180 on POD 3 as independent factors for CR POPF. A long operation time as a risk factor has also been demonstrated by others [9, 21]. A high level of CRP on POD 3 or an increase of CRP from POD 2 to POD 3 have been found as predictive factors [48]. The influence of ASA-PS on POPF is not clear. One explanation could be that elderly people are more likely to have some degree of atrophy in the pancreas, which could be related to a decreased POPF rate. Contrarily, elevated ASA-PS and increasing MPD diameter have been documented as risk factors by others [6, 49]. A high BMI, smoking, benign disease, younger age, male sex, and intraoperative blood loss have been associated with increased risk [3, 4, 9, 12, 48], but were not confirmed as risk factors in the present series. The ISGPS has emphasized the importance of a reliable risk score, and two scoring systems have recently been suggested [14, 49, 50].

PPH is the most serious complication after CR POPF and linked to mortality [12]. Also in the present study, PPH was more frequent in the CR POPF group with a high rate (5/17) in patients selected to ET. Although other published patient cohorts are small, PPH was not described in other series using ET [25–28]. This may indicate a different selection of patients to ET. The fatality in our ET group was caused by PPH, the patient underwent angiography and laparotomy. Three other patients successfully underwent angiographic interventions, while bleeding stopped spontaneously in the fifth patient with PPH. Thus, there is need for a multimodality treatment, including additional abdominal drainage which was frequently used in the ET group.

A limitation in the current study is the retrospective design. Interpretation of results are hampered by the lack of strict indications for ET, and non-standardized therapy (timing, procedure details, reinterventions, duration of stents). A strength of the study is the consecutive nature, all patients who underwent DP were analyzed, including a complete follow-up. The number of patients treated was small, but still we have not found any larger study and reports of ET of POPF are also scarce.

## Conclusions

CR POPF is a common and serious complication after DP with an often long healing time. As in previous series with fewer patients, the present study indicates that ET seems safe as complement to standard treatment of POPF but, without any obvious benefit. These findings should however be interpreted with caution due to small sample size, risk of selection bias, and lack of standardized treatment. Further studies are needed preferable in a prospective setting.

## Data Availability

Data may be available on request from the corresponding author.

## Abbreviations

DP: Distal pancreatectomy
CR: Clinically relevant
POPF: Postoperative pancreatic fistula
DGE: Delayed gastric emptying
PPH: Post-pancreatectomy hemorrhage
MPD: Main pancreatic duct
ET: Endoscopic treatment (ET)
EPS: Endoscopic pancreatic sphincterotomy
BMI: Body mass index (BMI)
ASA-PS: American Society of Anesthesiologists – Physical Status
ISGPS: International Study Group for Pancreatic Surgery
PV/SMV: Portal vein/superior mesenteric vein
CT: Computerized tomography
POD: Postoperative day
PPAP: Post-pancreatectomy acute pancreatitis
CRP: C-reactive protein
IQR: Interquartile range
ORs: Odds ratios
CIs: Confidence intervals
